# Factors underpinning the adoption of a school-based growth mindset intervention: a qualitative study

**DOI:** 10.1080/02667363.2024.2414455

**Published:** 2024-10-10

**Authors:** Kelly Morgan, Samantha Garay, Hayley Reed, Frank de Vocht, Simon Murphy

**Affiliations:** a Centre for Development, Evaluation, Complexity and Implementation in Public Health Improvement (DECIPHer), Cardiff University, Cardiff, UK; b Centre for Public Health, Population Health Sciences, Bristol Medical School, University of Bristol, Bristol, UK; c National Institute for Health Research, Applied Research Collaboration West (NIHR ARC West), Bristol, UK

**Keywords:** Growth mindset, intervention, children, education, educational psychology, implementation science

## Abstract

This qualitative study explores the motivations, barriers, and facilitators underpinning the adoption of the Mindset Teams programme in primary schools across Scotland. Semi-structured interviews were conducted with 18 teachers across six Mindset Teams schools and 14 wider stakeholders working across local, regional, and national levels. Findings suggested underpinning factors across the socio-ecological model, with differential themes identified, including six supporting school motivations and ten spanning barriers and facilitators, across teacher and stakeholder data. Limitations, implications for school and educational psychology (EP) practice, and suggestions for future school-based mindset interventions are considered.

## Introduction

Schools are considered ideal settings for the delivery of educational and health-promoting interventions, providing continued access to a diverse population of pupils. In 2021, the World Health Organization launched new global standards for health-promoting schools, encouraging all schools to nurture education and health and promote skills that foster healthy, prosperous futures for children (World Health Organisation, 2021). Central components of the Health Promoting Schools (HPS) model (Langford et al., 2014) are the development of simple policies to guide school activities and strengthen interactions between the school and nearby community. Despite the growing evidence-base for the potential of school-based interventions, there is often a reluctance for schools to adopt new interventions, with increasing concerns over workloads and poor-wellbeing among teachers (Grant et al., 2019; Santor & Bagnell, 2012; See et al., 2020; Skaalvik & Skaalvik, 2015). Furthermore, of the interventions which schools do decide to adopt, an estimated 50–75% of these are not implemented with sufficient fidelity (Gottfredson & Gottfredson, 2002; Rojas-Andrade & Bahamondes, 2019).

In recent years, there has been a surge in the number of school-based mindset interventions. Mindset interventions are programmes which aim to instil the belief that people can develop their attributes, abilities, and traits (Yeager & Dweck, 2012). Individuals with a growth mindset believe that attributes and abilities are malleable and can be improved. In comparison, individuals with a fixed-mindset believe that attributes and abilities are relatively stable or fixed (Blackwell et al., 2007). The evidence base around school-based mindset interventions is accumulating, with recent meta-analyses and systematic reviews demonstrating positive impacts on students’ academic achievement and mental health (Burnette et al., 2023; Jiang et al., 2024). A further review and meta-analyses, however, aired caution around the robustness of evidence of intervention impacts on academic achievement, revealing flaws in study designs, interpretation of results, and biases (Macnamara & Burgoyne, 2022).

School-based mindset interventions take many forms, with differing intervention durations, components, and delivery agents. Macnamara and Burgoyne’s (2022) systematic literature review and meta-analysis suggested that some interventions involve brief mindset practices, while others contain multiple interactive sessions spanning several months. Some further interventions were delivered face-to-face by teachers or researchers, and others use a combination of delivery agents and modes (Macnamara & Burgoyne, 2022). In Scotland, a teacher-led mindset intervention, “the Mindset Teams programme” (Winning Scotland, 2018) was introduced into schools in 2018 with an overarching aim to promote a growth mindset culture within schools. Recent analyses of school-level attainment data indicated positive programme impacts of the Mindset Teams programme on pupil writing scores, and analyses of teacher survey data revealed positive outcomes for teacher knowledge, attitudes, and beliefs (De Vocht et al., 2024). While a systematic review examined fidelity measures across mindset interventions in varying settings, there have been calls for more rigorous implementation studies (Savvides & Bond, 2021). To the best of the researchers’ knowledge, no study to date has reported on factors underpinning the adoption of a school-based mindset intervention in the primary school setting.

Literature highlights the complex nature of interventions within the school setting, with implementation requiring the active engagement of several factors, for example, teacher and student engagement (Bartholomew et al., 2011; Hall et al., 2011), adaptation to local contexts (Schaap et al., 2018), and consideration for the wider education and health system (Pearson et al., 2015). It is important therefore to consider the context and the broader ecological system in which school-based interventions operate. For instance, wider stakeholders (that is, individuals across wider parts of systems) have been shown to play a significant role during the implementation of an intervention (Rabin et al., 2008; Trompette et al., 2014; Walugembe et al., 2019) particularly across the macro-level of the social-ecological framework (Bronfenbrenner, 2005). This is depicted in mindset interventions whereby teachers, a key ecological asset, are not only a key intervention component but often the delivery agents (Futch Ehrlich et al., 2016). Campbell and Green (2022) emphasise the substantial lack of current ecological understanding around school environments and children’s mindsets, with an evident need to consider multiple stakeholder perspectives within research studies.

Implementation can be considered in three stages: adoption (that is, the decision to take-up the intervention); implementation (the delivery of the intervention); and sustainability (continuing to deliver the intervention after initial implementation) (Fixsen et al., 2005). Understanding which factors support or hinder each of these stages is helpful for intervention developers and funders, for those implementing the intervention, and those contemplating intervention adoption (Glasgow et al., 1999). Previous research highlights two broad areas affecting a school’s willingness and ability to adopt a new programme: the characteristics of teachers, for example, beliefs and attitudes towards the programme; and contextual factors of the school, for example, the level of administration support and leadership team buy-in (Beets et al., 2008; Chen, 1998). Based on theory, the Diffusions of Innovations model (Rogers, 1995) depicts the process by which members of a social system learn about, decide on, and adopt ideas or practices that are new. This theory highlights adoption as a staged process comprising awareness, decision to adopt or reject, implementation, and maintained use. Factors affecting the implementation (Domitrovich et al., 2008; Lyon et al., 2018; Pearson et al., 2015) and sustainability (Herlitz et al., 2020) of school-based interventions receive increasing attention, yet less consideration has been given to the factors underlying the adoption of interventions.

## Current study and research questions

Before paying attention to programme delivery and sustainability, a better understanding is needed of *why* schools decide to participate in a mindset intervention, and what factors help or hinder programme adoption. Such information is important for supporting the adoption of an evidence-based programme shown to have an impact on both pupils and teachers alike. This study reports on two aims: first, to explore motivations for schools adopting the Mindset Teams programme; and second, to examine the facilitators and barriers to programme adoption among schools.

Therefore, the underpinning research questions were:

- Why do schools adopt the Mindset Teams programme?
- What factors facilitate schools in adopting the programme?
- What factors act as barriers to schools adopting the programme?

## Methods

### Mindset teams intervention programme

The school-based programme consists of a 12-month blended learning journey, whereby a team of teachers undertook a 6-month online training course followed by a dedicated 6-month implementation period. Key areas of programme learning include understanding growth mindset, understanding school context, and applying mindset in one’s teaching. It is recommended that teachers dedicate 2–3 h per week for the online training, working through materials at their own pace. During the 6-month implementation period, teachers apply their acquired knowledge and skills to the school and classroom setting following a trying, testing, and reflecting process.

Within each school, teachers enrolled in the programme form a Mindset Team, typically comprising three members of staff, a Mindset Leader (senior management member) and two Mindset Champions (teaching practitioners). The formation of the Mindset Team is to ensure the growth mindset is implemented both at the classroom-level and more strategically at the whole school-level. Mindset Teams are provided with one-to-one tutor support throughout the programme and peer support is also available through an online response forum which is supported by Mindset Ambassadors, that is, teachers who have completed the scheme previously. Upon completion of the full programme, teachers are awarded 15 credits at Masters level.

The programme is offered to both primary and secondary schools by the programme provider, “Winning Scotland”. As of June 2023, over 676 primary and secondary schools in Scotland, comprising 29% of all mainstream schools (British Educational Suppliers Association, 2021) had undertaken the programme.

### Study context

This paper describes and evaluates qualitative data collected as part of a wider evaluation study. Data collection took place between September 2021 and July 2022. From programme inception to August 2021, a total of 150 primary schools had commenced the programme (24 of these started during the COVID-19 pandemic).

### Participants

The sample for this study included teaching staff at Mindset Teams primary schools (maximum of four teachers per school) and individuals (referred to as wider stakeholders) who had been involved in programme development or provision, secondary-school delivery, funding decisions or the wider policy arena.

A total of 137 mindset Teams schools were sent an email invitation to express an interest in participating in the study. Within each participating school, staff who had undertaken the Mindset Teams training programme were invited to participate. Using convenience sampling, a total of 48 wider stakeholders were sent an email invitation to participate in the study. Stakeholders were identified via the programme provider, Winning Scotland, and policy contacts were sourced with publicly available information.

Of the 137 schools sent a study invite, 91 (66.4%) did not respond, 35 (25.5%) declined and 10 (7.3%) expressed an interest in participating. A total of six schools participated in the research, all of which had commenced the programme in 2021. Schools were located across four local authority areas with varying levels of deprivation (Scottish Government, 2020). Across the six schools, a total of 18 school staff took part (including six headteachers). Table 1 shows an overview of school characteristics.

**Table 1.** Characteristics of schools participating in the study.

School ID	School Population	Deprivation	Location	No. of staff interviews
001	400-450	Medium	Accessible rural	4
002	200-250	High	Accessible small town	4
003	800-850	Low	Urban	4
004	100-150	Medium	Remote rural	1
005	200-250	Medium	Remote rural	3
006	150-200	High	Urban	2

Of the 48 wider stakeholders invited to participate, 28 (58.3%) did not respond, 6 declined (12.5%) and 14 (29.2%) took part in an interview. Participants included four programme providers, two local authority education officers and four national stakeholders. Four secondary school representatives (a headteacher and three teachers) were also invited to participate as wider stakeholders as it was considered important to capture insights from the broader educational setting. To provide participant exemplar quotes, the following ID key has been used: HT = headteacher, MC = mindset Champion, ML = mindset Leader, WS = Wider stakeholder, with accompanying numbers showing a participant ID or group number.

### Data collection

One-on-one semi-structured interviews were conducted (researchers HR and KM) with participants via telephone or virtually using Teams, depending on the discretion of the participant. Interviews lasted between 25 and 65 minutes. Topic guides, co-developed with the study advisory group and teachers at the study’s Public Involvement school, were used to explore key programme areas: motivations and adoption; implementation; sustainability, and perceived impacts on children and teachers. All participants provided written and/or audio consent for participation; audio recording and use of anonymised quotations. The Consolidated criteria for Reporting Qualitative research checklist (COREQ) (Booth et al., 2014) was consulted for reporting the study. A sample of the semi-structured interview schedule is appended (See Appendix 1).

### Data analysis

Data were analysed by three female researchers, KM, HR and SG, using verbatim transcriptions and NVivo 12 software. Following initial review checks, data were coded inductively following the reflective thematic analysis approach of Braun and Clarke (Braun & Clarke, 2006). To ensure trustworthiness of the data consistent methods and measures within data collections were employed, and of a third of data transcripts were double-coded. For each dataset, a conceptual framework was produced to include initial themes and sub-themes. This process involved researchers reading, open coding, and discussing a small number of transcripts before agreeing any amendments to the coding framework. Upon agreement, a systematic approach to data management was adopted, with all transcripts coded into the framework.

Following the completion of coding, a detailed overview of participant data populating each theme and sub-theme was created. Findings were compared across schools and participant groupings, with areas of difference highlighted in the results. The socio-ecological model (Bronfenbrenner, 2005) was used as a framework for displaying findings of adoption barriers and facilitators across different levels of the model. Participants were provided with a summary of the overall research findings.

## Findings

For each research aim, key themes and accompanying exemplar quotes will be described below.

### Aim 1: explore school motivations for programme adoption

**Figure 1.**
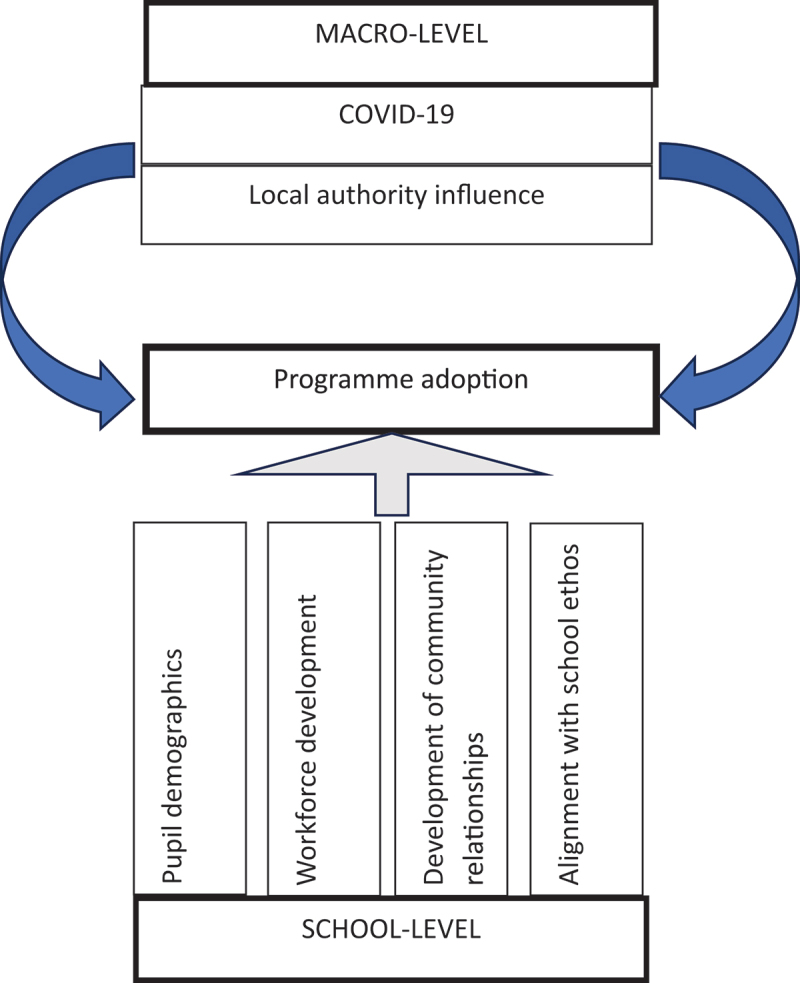
Depiction of key themes influencing programme uptake by schools.

When describing the underlying motivations for taking part in the Mindset Teams programme, school motivations spanned school-level factors and macro-level factors (see Figure 1). School-level factors included four themes, pupil demographics (for example, socio-economic status), workforce development (for example, personal development and leadership planning), alignment with school ethos (for example, supporting existing efforts, delivery of school improvement plans), and the development of community relationships. Macro-level factors underlying programme adoption included two themes, local authority influence and COVID-19.

## School-level factors

### Pupil demographics

Among four schools, several teachers spoke about pupil demographics influencing their decision to take up the programme. In three of these schools, teachers described how deprivation was directly linked to social and emotional problems among pupils. For some, the programme was seen as a way of improving pupil behaviour or tackling high anxieties around certain subjects, for example, maths was commonly mentioned. Many teachers, however, frequently described how the programme was adopted to combat pupils’ low aspirations, with an evident lack of role models and supporting family aspirations.

I think a lot of children, and I’ve heard them say this, “like I’m from this certain street so I’m not going to do anything like that I’m not going to get very far”. So there’s certain places within [Area named] that’s even lower than that Street and that’s kind of the area where you don’t want to be from. So there’s a lot of negative mindset that comes even from just the area itself. (002 MC2)

Contrastingly, in one school, teachers spoke of the area’s high affluence levels and how there was an identified need to improve pupils’ resilience levels, despite the common misconceptions around pupil affluence and resilience.

We’ve had a lot of kind of conversations in years gone by about resilience and about building children’s resilience and building their attitude towards – we are quite an affluent authority. So the presumption is that we have all these highly able children, but it can be – they’re maybe adverse to a bit of challenge. So, we’ve talked a lot about building resilience and we’ve tried things … to build up resilience. (003 MC1)

### Workforce development

#### Personal development

There were several instances whereby schools had largely taken up the programme due to the influence of senior leaders. Some headteachers noted how programme adoption was due to their personal interest in the topic or a recognised need to develop their own growth mindset.

I guess for myself, from my own point of view, I have this thing that I sometimes, it’s not that I give up but I get quite down if things aren’t quite going the way that I expected them so even for myself, learning about growth mindset and developing that within myself as well as within the children was really quite important. (005 HT)

#### Leadership planning

Often, the motivation for personal development was coupled with an eagerness to empower wider staff members. In two instances, teachers spoke of their motivation to undertake additional professional training and how this programme had been identified as beneficial to pupils within the schools.

It was also an opportunity for myself to take part in another course. I just finished an online university course, but it [Mindset Teams programme] allowed that empowerment of members of staff as well – so I’m very keen on staff members having the opportunity to lead in different areas, so this gave [Teacher named], an opportunity to lead and be part of it as well. (004 HT)

### Alignment with school ethos

#### Supporting existing efforts

Most school staff described how their school had already undertaken some form of growth mindset work to support pupils. Former growth mindset work had largely encompassed light touch activities informed by teachers’ wider reading. As such, the adoption of the Mindset Teams programme was often highlighted as a way to introduce a more structured, formal practice within schools.

It was something that we’ve always looked at, growth mindset we’ve kind of scratched the surface here but it was the fact it was a proper programme and it would improve our wellbeing as well as teachers for CPD and just a lot more structure for us so I think that’s what we found quite interesting. (005 MC1)

#### Delivery of school improvement plans

Growth mindset had been identified as a priority area for most schools prior to programme adoption, with many teachers highlighting how the Mindset Teams programme was perceived as pivotal to the delivery of School Improvement Plans. Teachers described that pupil health and wellbeing were identified as a key focus within plans and that Mindset Teams was perceived as a key vehicle to achieving this.

Wellbeing is a massive part of our School Improvement Plan, so it’s got its own priority and the children’s mental health and how they focus and look at themselves. That’s quite a target and quite a challenge that they work towards as well and the growth mindset course just all tied in quite well. We could see that there was a space and a need for it and we were really putting a lot of faith in it. (002 MC1)

### Development of community relationships

In three schools, teachers highlighted that a key motivation for taking up the programme was to build much-needed connections with families and wider communities. Teachers described how they had identified a need to increase engagement, with one teacher highlighting this as an avenue to improve the lack of pupil aspirations stemming from the home environment. For another teacher, increasing the school’s engagement with parents following COVID-19 was noted as particularly important.

Because parental engagement is something we’ve identified as a school post lockdown that’s really important … we need to get our parents back into class, back into school to see what their kids are doing and also to – the next stage is sharing. Embedding first of all the practice, that growth mindset and resilience across the school, but also sharing with parents so that they can work in partnership with them so that the message is consistent at home and at school. This is what we do at school to help with maths. This is what you can do at home as well. (001 HT)

## Macro-level factors

### Local authority influence

In one school, programme adoption was underpinned by a priority call from the local authority. A teacher described how growth mindset had previously been on the school agenda, but in more recent years, this had no longer been a priority due to several barriers (for example, staffing changes, COVID-19). The local authority call was therefore viewed as a positive step from the school’s headteacher to re-engage with growth mindset efforts and seen as a training opportunity for the teacher.

The local authority had sent them [Headteacher] an email and had requested members to be part of it. I was already considering doing some form of study, so I decided to do that instead. (006 MC1)

### COVID-19

Most teachers mentioned the role of COVID-19 in reinforcing the need for programme adoption. Teachers described how the pandemic had impacted on children’s wellbeing, in particular, creating low morale. The programme was seen as important for supporting children’s social and emotional wellbeing and especially combating any compounding impacts from the pandemic.

From coming out of the pandemic … I mean we still are working through it, but noticing I suppose especially with some of the younger kids who have not really been in school at all much, kind of resilience in their learning and sort of their engagement with challenge. (003 MC2)

## Aim 2: examine the facilitators and barriers to programme adoption

**Figure 2.**
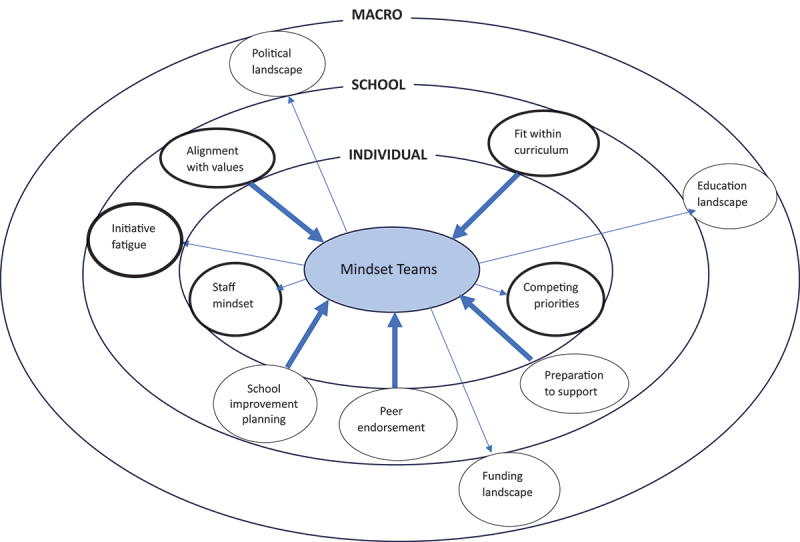
Uptake of the mindset teams programme – facilitators and barriers shown across a socio-ecological model (bold arrows depict facilitators and narrow arrows depict uptake barriers. Bold circles indicate themes highlighted by teachers and wider stakeholders).

Facilitators and barriers to programme adoption were discussed among teacher and wider stakeholder data. Identified themes spanned three levels; the individual-level, the school-level, and the macro-level. The individual-level comprised two themes; staff mindset and competing priorities (both identified across both datasets). The school-level encompassed five themes; embedded within the school system, alignment with values, preparation to support teachers, initiative fatigue and peer endorsement. The first two themes were identified within both datasets. The macro-level comprised three themes, all of which were identified in the wider stakeholder data only; political landscape, education landscape and funding landscape. Figure 2 displays each theme across a socio-ecological model depicting themes identified as adoption facilitators and those themes identified as adoption barriers.

## Individual level

### Staff mindset (barrier)

Teachers talked about the barrier of some staff members having a fixed mindset. This was largely described in relation to the wider staff population among schools. Programme adoption was hindered when staff members did not see the importance of the programme and staff were therefore unlikely to engage with the programme and invest time into the training. One teacher spoke about their perception that some staff might consider themselves to already have a growth mindset having done some training a few years ago, yet the teachers emphasised how there was a clear need for growth mindset to be viewed as a long-term investment.

… you know that false mindset, we’ve done a growth mindset, we’ve got growth mindset, we did that two years ago so it’s done, it’s finished, you know, that lack of understanding that it is a methodology, it’s a pedagogy, it’s not a tick your box and we’ve done and we’re all there, you know, it’s not a destination, it is about a process … (006 MC2)

Among wider stakeholder interviews, many participants also discussed a lack of staff understanding around mindset as a barrier to programme adoption. In one instance, a wider stakeholder describes how this lack of understanding is linked to a perception that the programme is not warranted at their school.

It’s just really about the culture or belief in children’s ability to learn and progress and challenge and everyone has built that potential, but I think in a naive way, most teachers, headteachers would probably say “oh we believe in all that as well” but then that’s not a full understanding. (WSI 6)

### Competing priorities (barrier)

Across most schools, teachers spoke of the programme’s time demands posing a barrier to adoption. This encompassed the high amount of time required for teachers to undertake the training amongst the ever-growing demands placed on teachers’ workload. This barrier was also acknowledged throughout wider stakeholder interviews, with a shared perception that schools often prioritise workloads which are directly linked to raising attainment. One teacher spoke of the direct implication for schools who do not have enough staff to cover lessons, while other staff undertake the training. As a result of the programme demands and conflicting high workloads, two teachers also emphasised the difficulty in sourcing teachers willing to undertake the training.

But I think other primaries might say well that’s a lot of work or it’s a lot of work to get our kids to change their kind of mindset it’s a lot of teaching. It’s now completely embedded throughout what we’re doing, and I think you can see that, but I think to get to that point it is a lot and I think people might be put off by that. But I think seeing the changes and seeing the difference it’s amazing that it’s been in our school it’s definitely worth it. (002 MC2)

As the study took place amidst COVID-19, many wider stakeholders and a few teachers stressed how schools were facing even greater competing demands. That said, some stakeholders highlighted their belief that schools’ interest in growth mindset had begun to fade, even before the pandemic, with their focus shifting towards other priorities such as inclusion. Teachers spoke about how the pandemic might have contributed to staff becoming uninterested and less willing to undertake the required training.

From my point of view some of the staff in our team weren’t too invested in it and maybe still not sold on the idea … because the children had missed so much school in the last two years the thought of giving up time to [Teacher named] and I our project we were introducing, juggling to learn a new skill together to get them to experience the aspects of growth mindset through juggling and from other teacher’s perspective. I think some of them thought we’re giving up teaching time to teach our children how to juggle, that’s not a skill they need for life sort of thing. (006 MC2)

## School level

### Embedded within the school system (facilitator)

#### Fit within curriculum

Both wider stakeholders and teachers acknowledged that the programme aligns with various school agendas and how this was an evident facilitator to adoption. There was recognition of the programme’s fit within the existing curriculum, with descriptions of its inclusive and complementary nature among other subjects. Wider stakeholders also relayed how the programme can adapt to the broader context (for example, using an action research methodology to evaluate what is making a difference).

There’s the perception out there that STEM is only for certain people. They talk about the Einstein theory that it’s white, crazy-haired scientists who are male, and yet STEM is, as we are saying, is actually for everyone. Sometimes people don’t understand, depending on their background or their experience. So Mindset’s programme can help explore some of that. (WSI 8)

One teacher stressed the importance of ensuring that schools understand *how* the programme is beneficial for schools alongside an understanding that the programme can be incorporated across all aspects of the curriculum. When describing the latter point, the teacher emphasised how the programme should not be viewed as an additional activity to fit into the busy school day. Another teacher described how this awareness became particularly apparent after they had completed the online aspect of the training course.

But I suppose that’s after you’ve done the course, you can see how you can just build into what you’re doing, which makes it easier because it’s not an add-on. But I think it’s so much of what we’re wanting, like the dispositions and things you want for learners now, it’s part of that. So I don’t know. I can’t understand. I don’t know why anybody wouldn’t want to do it, to be honest. (003 MC1)

#### School improvement planning – autonomy

Some headteachers recognised the importance of having autonomy over the areas included within the School Improvement Plan. Teachers relayed how historically content had been largely determined by the local authority, which was perceived as a key barrier to programme adoption. In more recent times, however, there had been an increasing focus on schools having ownership over their own School Improvement Plans to ensure that plans were fit for their community and context.

It’s about making that a priority as part of your School Improvement Plan. So another barrier is just what individual schools have in the School Improvement Plan. The past couple of years our School Improvement Plan has been very much authority led because we’re all working on the same outcomes. It was about digital, it was about improving pedagogy, coming back and it was about improving health and wellbeing. Whereas now this year we’re all doing our own priorities now based on our school evaluation. (002 HT)

#### School improvement planning – key focus

Across the majority of discussions, there was recognition that including growth mindset within the School Improvement Plan was a clear facilitator to adoption. Teachers described how this written commitment ensured growth mindset was seen as a priority and focal area, which evidentially facilitated the programme being introduced within the school. While acknowledging the competing demands for including items within School Improvement Plans, teachers acknowledged how the Mindset Teams programme is viewed as an all-encompassing piece of work which is cross-cutting of numerous subjects.

What happens at this time of year is that everybody wants to throw their own agendas in on top of your School Improvement Plan … You know people saying come in and say well, we’ll unlock funding for you if you put the sciences in your School Improvement Plan. We’ll unlock funding for you if you put modern languages explicitly in your School Improvement Plan. Now, we’ve resisted those things because it’s not that we don’t think that they’re important, but we sort of have an eye on addressing some of the bigger things. And I think Growth Mindset is applicable in the sciences and in modern languages. (003 HT)

### Alignment with values (facilitator)

A key perceived facilitator to programme adoption was the alignment of the programme with personal and professional values, not only at the teacher level but across the senior leadership team and whole school. Throughout interviews, teachers described the importance of shared values as a school and having buy-in as a senior leadership team to support programme adoption. Wider stakeholders also acknowledged the influence of key individuals across local authorities who can advocate for the programme. In some instances, these individuals were described as valuing the programme highly due to the results they had seen by changing mindsets.

Across certain authorities – there are definitely people who, I suppose, if they can see the impact and really believe in “Mindset” themselves – not even from a local authority perspective, but even professional – a personal – level, then they understand, or are backing it themselves – that enthusiasm probably rubs off, and it definitely has helped. (WSI 1)

However, teachers described instances whereby staff were not invested in the programme or could not see the value of the programme, which was considered a key barrier to adoption.

I think we were fortunate in the sense that we did have that personal interest as well it kind of motivated our staff but not all staff and not all schools are going to have that until they start, and they buy into it themselves and then they’re engaged. (002 ML1)

The Mindset Teams programme was viewed by some teachers as a natural fit for supporting the development of more holistic skills such as problem solving and creativity as opposed to the historic focus on traditional curriculum teaching. One headteacher emphasised the importance of school values not focusing purely on children’s academic achievement but instead ensuring that values place greater importance on supporting children more holistically. Supporting a growth mindset among pupils was considered key to this focus.

But I just think that comes back down to your values ultimately as a school … I think sometimes … the value or the worth of a school or the effectiveness of a school can sometimes be judged in quite a one-dimensional way. “How good’s your attainment?” Well, we have a set of values in here that says, well actually, it’s about much more than that. (003 HT)

### Preparation to support teachers (facilitator)

Within half of the schools, teachers described the importance of undertaking appropriate planning to support programme adoption. For some, this included the need to allow sufficient time allocation to enable teachers to undertake the training course and facilitate discussions among school teams. Being upfront about the required workload was considered a key part of this preparation. One teacher spoke about the need for clear planning and communication to avoid any confusion around training course assessments, for example ensuring schools are aware of the time requirements for completing the course. One teacher also highlighted the importance of being organised to ensure the programme was included in the School Improvement Plan in a timely manner.

… it would just be timing and working out with your school how it was going to work, like a good plan before it started, rather than saying you’re doing this course. Our school was very good at laying out exactly what it would involve, how many hours it would take, what would be expected of us, it was all laid out before that. So I would say if people got the information prior to it all starting. (001 MC2)

### Initiative fatigue (barrier)

Closely linked to the theme of competing interests, wider stakeholders (programme providers and national stakeholders) recognised that the existence of multiple school initiatives can present a barrier to schools uptaking the programme. Stakeholders mentioned that schools are frequently offered several initiatives at once, creating a competition for school attention, while schools have already implemented other approaches. One stakeholder described how this large volume of initiatives leads to poor implementation in schools.

We sometimes feel like there’s always something being introduced, and these things aren’t followed through on the ground in schools. So, whether that’s a growth mindset, or whatever you want to say, it’s something new that’s introduced, and then it doesn’t … they’re very wary of introducing something else. (WSI 3)

Some alternative initiatives considered complementary to the Mindset Teams programme, for example, those also focusing efforts on closing the attainment gap, were however, described as possible facilitators to programme adoption. Conversely, stakeholders acknowledged that several competing mindset-focused initiatives exist, some of which offer similar content or delivery style. In this instance, stakeholders highlighted how it can be difficult for schools to know into which programmes they should invest time and resources.

So it might be that a particular school, for example, has not particularly good attainment in numeracy and that is what they are focusing on and not looking at growth mindset because there is real pressure to improve numeracy. Now, I know that if you do both the numeracy project and growth mindset you will probably get better results, but I am not a headteacher in every school and so you can see what I am driving at. (WSI 11)

### Peer endorsement (facilitator)

Within interviews, programme providers discussed the role of other schools in supporting programme adoption. It was acknowledged that personal recommendations for the programme from one school to another were a key facilitator to adoption. One stakeholder described how peer endorsement could be a powerful tool for overcoming barriers related to initiative fatigue.

Absolutely, because that’s what swings it for them time and time again. Peer to peer is very empowering because teachers love direct recommendations because, as I say, they have initiative fatigue, but they love it and they know that if something that people have tried and it’s had a very positive impact and so schools will be much quicker to adopt something. (WSI 2)

## Macro level factors

### Political landscape (barrier)

Among wider stakeholder discussions, national and local-level stakeholders identified the current political landscape as a barrier to programme adoption. Stakeholders described how the fragmented landscape makes it difficult to diffuse innovative concepts throughout schools. This was often discussed with examples of a lack of joined-up thinking, high staff mobility across schools and inconsistent leadership.

… at a political level Scotland’s a small country, but it’s got 32 local authorities. That’s 32 directors of education, 32 sets of elected members, and they don’t all work as well. Education Scotland can bring forward initiatives, but at the moment that’s normally being driven by post-inspection where there have been issues, so people need to have a plan about how they’re going to improve things. It’s very difficult to get 32 local authorities all doing the same thing. (WSI 10)

### Funding landscape (barrier)

Stakeholders highlighted a lack of clarity around programme funding as an adoption barrier, with particular emphasis on the uncertain funding source and the funding provider. For example, it was unknown whether responsibility was placed with individual schools, the local authority or national policy-makers. A few stakeholders, however, suggested that this barrier had decreased as “evidence” of programme impact had developed.

I think the whole funding landscape is so complicated. Pots of money one year are not there the next year, whether it’s pupil equity funding or whatever, so it comes down to not just who the customer is, but ultimately where’s the pot of money coming from? I think those that are really passionate about Mindset Teams and about growth mindset more generally are desperately trying to shoehorn that concept into a pot of money and make it happen. (WSI 7)

Within some local authorities, there was an evident steer on dissuading schools to adopt the Mindset Teams programme. Across stakeholder interviews, discussions highlighted how some authorities opted to invest in their own development teams or to fund an external provider to deliver growth mindset training in-house. Some authorities were known for actively discouraging schools from going to outside agencies.

Well, anecdotally, from conversations with teachers within schools and having been working at local authority level myself, local authorities have a range of priorities that they focus on and a number of them have indicated that they have engaged with the growth mindset from within their own cohort of practitioners, particularly if a local authority has a pedagogy team identified within their services. (WSI 2)

### Education landscape (barrier)

National-level stakeholders expressed a perceived disillusionment among the teaching workforce. This was viewed as an adoption barrier, with stakeholders describing a lack of engagement in staff training, leading to a lack of staff enthusiasm, in addition to the younger workforce potentially becoming stifled by the pressure to do things the “way they are always done”.

There’s been a lack of consistent almost like vocational training throughout the education system to keep teachers engaged, to keep them enthusiastic, to keep them motivated. I genuinely believe teachers go into teaching for the best of reasons, almost like politicians to be honest, and I do believe that, but I have seen and am seeing, … … friends in the education system and some of them are already getting disillusioned in the state sector by the constant paperwork, the constant edicts from above. (WSI 14)

## Discussion

To the best of the knowledge of the authors of this paper, this is the first study to explore factors underpinning the adoption of a school-based mindset intervention. The socio-ecological model (Bronfenbrenner, 2005) was used as a framework to display findings, which provided a basis for understanding how barriers and facilitators are patterned across different levels. Throughout analyses, it was possible to identify similar and contrasting views between school staff and wider stakeholders.

Motivating drivers for the adoption of the Mindset Teams programme were largely found at the school-level, with themes centred on supporting the needs of the pupil demographic and the development of the school workforce. There was also significant recognition that most schools had sought to adopt the programme because Mindset had been identified as an existing area of work or a focused-need within strategic school plans. This finding is in line with prior work (Pegram et al., 2022), highlighting that schools’ decision-making around intervention selection is typically influenced by a programme’s compatibility with past practices, with some studies further indicating that decision-making is often irrespective of the quality or quantity of available supporting evidence (Brown & Greany, 2018; Hallfors & Godette, 2002).

The Diffusion of Innovation Theory (Rogers, 1995) highlights the importance of programme compatibility with the values, needs and experiences of programme adopters. Within the current study, programme alignment with values and the school curriculum was also identified as a key facilitator to adoption. Both teachers and wider stakeholders recognised shared values among school leaders, the school workforce and surrounding local authority as key to ensuring a successful joined-up approach to programme adoption. Pearson et al. (2015) highlight that active support by senior figures is instrumental in ensuring that not only is a new programme embedded within written school policies but that the programme is also actioned on the ground, which echoes teachers’ views within the current study. There was a clear emphasis on the need to undertake actions to prepare expectations of the workforce (for example, transparency regarding the amount of work required) and ensure sufficient support was in place (for example, time allocation and staff cover). One action, identified as a key uptake facilitator among schools, was the use of a written commitment to growth mindset within School Improvement Plans. Wider studies suggest that written commitments can ensure staff accountability to the programme (Tjomsland et al., 2009) and can also help combat issues related to staff turnover (Tjomsland et al., 2009), for example, making a programme resilient and sustainable (Dijkman et al., 2015)).

A total of six programme uptake barriers were identified. Consistent with other literature, two of these barriers were teacher mindset and competing interests and were identified across teacher and wider stakeholder data. There was a recognised concern around teachers’ mindset and programme buy-in, with a perceived lack of teacher understanding or recognition around the programme need. There were clear examples in the data whereby individuals recognised fixed mindsets among colleagues, and these were identified as a key barrier to programme uptake. According to the mindset -supportive-context hypothesis, the role of teacher mindset is crucial for the delivery of growth mindset interventions, suggesting that teachers with a fixed-mindset are more likely to create a classroom environment which does not support growth mindsets among pupils. This hypothesis was supported by recent findings from a US-based study examining mindsets among maths teachers (Yeager et al., 2022).

An important contextual barrier in the present study was the role of initiative fatigue in schools, a theme closely linked with the identified barrier of competing interests. Both teachers and wider stakeholders acknowledged the increasing demands placed on the teaching workforce and the sheer number of programmes that schools are presented with. Some teachers emphasised these barriers in the light of the intensive time demand posed by the Mindset Teams training requirement. Woven throughout these discussions also was the impact of COVID-19, with all participating schools adopting the programme amidst the pandemic. Teachers recognised that the pandemic had created additional workload pressures and competing priorities and speculated that this might have also contributed to some programme uninterest among staff. However, some stakeholders believed that school priorities had shifted to other areas of pupil learning and wellbeing before the onset of the pandemic. The role of the pandemic is an important consideration when viewing all findings of the current study.

A clear difference in the perspectives of school staff and wider stakeholders emerged when considering influencing factors within the macro-context. While teacher accounts spanned the individual and school-level only, wider stakeholders identified three adoption barriers within the macro-context. Barriers concerned the funding, political, and educational landscape in Scotland, with stakeholders emphasising the realisation that schools are complex adaptive systems working with and within an evolving context (Keshavarz et al., 2010). Stakeholders described the fragmented legislative and leadership approaches across the 32 local authorities in Scotland, with the political set-up perceived to be hampering the diffusion of innovation among schools. There was a perception that initiatives were often introduced as a reactive response to school inspection as opposed to school innovation and that this way of working did not support a unified approach to the Mindset Teams programme.

Entwined within discussions was also the perception that a lack of coherence around funding streams (that is, where programme funding should come from), and funder responsibility (at what level the decision on funding provision should be made) was creating a further barrier to programme adoption. Racine (2006) argues that programmes or innovations are more likely to be successful when they are favoured by their socio-political environments; for example, by the individuals, organisations, and wider influencers that shape rules and expectations. Racine further argues that programmes are unlikely to reach full potential unless there is acceptance within the political arena, given its influence over public policy and funds.

Consistent with other literature, national stakeholders recognised the resistance among the teaching workforce to undertake vocational training and described this as an inherent problem of the education system in Scotland. This issue has previously been identified for teachers working within impoverished actor networks, such as those experiencing high workloads, time constraints and part-time contracts (Broad, 2015; Sappa et al., 2019). While not identified as a macro-level barrier by teachers, this barrier is tangential to teacher discussions around staff mindset and competing priorities, signifying how programme adoption is largely underpinned by the receptiveness of school staff.

In Scotland, educational psychologists (EPs) work closely with schools to support children’s attainment and health and wellbeing (The Association of Scottish Principal Educational Psychologist, 2024). The majority of EPs contribute to the development of education authority guidance and policy for schools. At the school-level, EPs help schools to link their school improvement plans to an action research approach, often supporting the planning of interventions. Education Scotland has identified EPs as an important conduit for supporting health and wellbeing approaches in schools such as growth mindset interventions (Education Scotland, 2019).

### Strengths and limitations of the current study

This study has drawn on a range of perspectives to explore factors underpinning the adoption of a growth mindset intervention in order to produce new insights which are relevant to multiple stakeholders. Several limitations of the study need to be considered. First, a small sample of schools participated in the current study (4.4% of eligible schools at the time of recruitment), all of which had adopted and implemented the programme. Data gathered from a larger sample, including non-adopting schools, might have yielded a different result. Second, with varying educational contexts across the UK (Maisuria, 2023) and globally, the transferability of findings requires consideration. Third, the social desirability bias of school participants and wider stakeholders cannot be ruled out. Fourth, this study took place amidst the COVID-19 pandemic during which schools experienced significantly heightened pressures. This had an evident impact on study recruitment, and it is important that findings are viewed in the light of the wider context.

## Conclusion and implications for EP practice

A multitude of factors facilitate and inhibit the adoption of school-based programmes. The current study unearthed such factors relating to a growth mindset programme implemented in schools in Scotland, with factors identified across the socio-ecological model. While some factors echo systemic challenges across the educational and political landscape, several highlight practical steps which can aid successful programme adoption. As such, findings are important for programme funders, providers and wider educational professionals (for example, EPs) to ensure effective implementation strategies are in place to support the adoption of an evidence-based growth mindset intervention, Mindset Teams, among schools.

Considering the current findings and wider literature, the following recommendations are offered to assist EPs in considering ways to support the adoption of growth mindset interventions, drawing on Mindset Teams as an example of real-life practice across Scotland. These recommendations are provided as helpful prompts, rather than as an exhaustive list:

- Support schools to ensure that school improvement plans and wider efforts are coordinated to support programme adoption and reduce initiative fatigue.
- Work with teachers (for example, Mindset Teams) to identify the professional learning needs of the wider school workforce.
- Offer expertise and insights to support strategic local authority-wide decisions on mindset-related services and funding.
- Support schools to strengthen relationships with the wider community, such as parents and carers.
- Advise schools on appropriate tools to help evaluate the implementation and impacts of the Mindset Teams programme on teachers and children.

The current study highlights important considerations for the adoption of school-based growth mindset interventions and provides practical recommendations for future implementation strategies.

## Appendix 1. Examples of semi-structured interview questions

1. How do health initiatives for primary schools come to be introduced and implemented?
2. How would you say schools/communities respond to the services, programmes and interventions offered?
3. What do you think affects whether approaches are taken up by schools/communities?
4. Why do you think Local Authorities have wanted to be involved in the programme/programmes about mindset?
5. What do you think has supported Local Authorities to uptake the Mindset Teams programme/programmes about mindset?
6. Some Local Authorities are yet to become involved in the programme, are you aware of any possible barriers to becoming involved in programmes about mindset?
7. For what reasons do you think primary schools have become involved and deliver the programme/programmes about mindset?
8. What factors support a primary school to uptake the Mindset Teams programme/programmes about mindset?
9. What factors could hinder or prevent a primary school to uptake the Mindset Teams programme/programmes about mindset?
